# Restriction factors in human retrovirus infections and the unprecedented case of CIITA as link of intrinsic and adaptive immunity against HTLV-1

**DOI:** 10.1186/s12977-019-0498-6

**Published:** 2019-11-29

**Authors:** Greta Forlani, Mariam Shallak, Elise Ramia, Alessandra Tedeschi, Roberto S. Accolla

**Affiliations:** Laboratories of General Pathology and Immunology “Giovanna Tosi”, Department of Medicine and Surgery, School of Medicine, University of Insubria, Via Ottorino Rossi 9, 21100 Varese, Italy

**Keywords:** Intrinsic immunity, Restriction factors, HTLV-1, HIV, CIITA, Tax

## Abstract

**Background:**

Immunity against pathogens evolved through complex mechanisms that only for sake of simplicity are defined as innate immunity and adaptive immunity. Indeed innate and adaptive immunity are strongly intertwined each other during evolution. The complexity is further increased by intrinsic mechanisms of immunity that rely on the action of intracellular molecules defined as restriction factors (RFs) that, particularly in virus infections, counteract the action of pathogen gene products acting at different steps of virus life cycle.

**Main body and conclusion:**

Here we provide an overview on the nature and the mode of action of restriction factors involved in retrovirus infection, particularly Human T Leukemia/Lymphoma Virus 1 (HTLV-1) infection. As it has been extensively studied by our group, special emphasis is given to the involvement of the MHC class II transactivator CIITA discovered in our laboratory as regulator of adaptive immunity and subsequently as restriction factor against HIV-1 and HTLV-1, a unique example of dual function linking adaptive and intrinsic immunity during evolution. We describe the multiple molecular mechanisms through which CIITA exerts its restriction on retroviruses. Of relevance, we review the unprecedented findings pointing to a concerted action of several restriction factors such as CIITA, TRIM22 and TRIM19/PML in synergizing against retroviral replication. Finally, as CIITA profoundly affects HTLV-1 replication by interacting and inhibiting the function of HTLV-1 Tax-1 molecule, the major viral product associated to the virus oncogenicity, we also put forward the hypothesis of CIITA as counteractor of HTLV-1-mediated cancer initiation.

## Background

Immunity against pathogens and pathogen adaptation to their host coevolved and keep evolving in symbiosis as a continuous process with mutualistic and antagonistic features to guarantee protection of the host as a species and selection of the pathogen for the best fitting without killing the host [1]. Mechanisms of host immunity have been classified in distinct forms depending on the main cell and molecule effectors involved. Innate immunity and adaptive immunity are the two major forms of defense in higher eukaryotes, acting mostly in non-specific and pathogen-specific way, respectively [2, 3]. Nevertheless, this distinction is rather artificial as cells and molecules of innate and adaptive immunity often cooperate each-other and actually in many cases they trigger each-other, again showing a concerted evolution for the protection of the host [4]. An additional form of immunity, designed intrinsic immunity, operates in parallel to the two major forms of protection and relies on intracellular molecules defined as restriction factors (RFs), either constitutively expressed or induced by mediators of innate immunity, whose function is to counteract distinct steps particularly of virus life cycle [5, 6]. As a reaction, viruses have evolved strategies to evade the antiviral activity of these host proteins, thus favoring viral infection and spreading. Due to these effective escape mechanisms, RFs are generally inactive at controlling viral replication in their natural host, however they are potent antiviral effectors against viruses from other species, thus play an important role in making species-specific barriers against viral infection [6–8]. Here we discuss current progress in studies of human retrovirus specific RFs, with special emphasis to those involved in HTLV-1 infection. An introduction on RFs against HIV-1 will precede the description of RFs and HTLV-1, because RFs were first described as counteractors of HIV-1 infection in order to compare their mechanisms of action with those described for HTLV-1.

## HIV-1 restriction factors

RFs were first identified as inhibitors of Human Immunodeficiency Virus 1 (HIV-1) infection, targeting various stages of viral life cycle, from capsid uncoating to viral budding [6, 9] (Table 1). HIV-1 has evolved a variety of strategies to overcome intrinsic immunity, mainly by using some viral accessory proteins, such as Viral Infectivity Factor (Vif), Viral Protein U (Vpu), or Negative Regulatory Factor (Nef) [6, 7, 10]. HIV-1 antiviral host factors such as Apolipoprotein B mRNA editing enzyme-catalytic polypeptide-like 3 (APOBEC3) family [11], Tripartite motif 5α (TRIM5α) [12, 13], tetherin/BST-2 [14, 15], and Sterile Alpha Motif and HD containing protein 1 (SAMHD1) [16] have been well studied with regard to the biological mechanism of the antiviral response [7]. APOBEC3G (A3G), identified as the first host restriction factor that potently inhibits HIV-1 infection [11, 17], is a cytidine deaminase loaded into the virus particle during assembly. A3G catalyzes cytosine-to-uracil deamination in the nascent viral DNA, generating a high frequency of G to A mutation and premature stop codons. The resulting defective proteins assemble non-functional viral particles, responsible for the potent inhibition of HIV-1 replication. Vif neutralizes the antiviral activity of A3G by inhibiting its packaging into viral particles and thus promoting its proteasomal degradation. In addition to inhibiting the replication of Vif-deficient HIV-1, A3G has been shown to inhibit the replication of other exogenous and endogenous retroviruses, retrotransposons, and Hepatitis B virus (HBV) [18–27].

**Table 1:** Restriction factors involved in human retroviruses infections

Restriction factors	Mechanism of restriction		
	HIV-1	HTLV-1	HTLV-2
APOBEC3G	Inhibits viral transcription of Vif deficient HIV-1, by generating G to A mutations on nascent viral DNA [17]^a^	No restriction [26]	nr
TRIM19/PML	Inhibits viral transcription [35]	Inhibits viral replication by targeting Tax-1 for proteasomal degradation [87]	Inhibits viral replication by targeting APH-2 for proteasomal degradation [40]
SAMHD-1	Inhibits viral transcription by depleting endogenous dNTP pool [16]	Induces apoptosis of HTLV-1 infected cells [90]	nr
Tetherin/BST2	Prevents viral particles release [15]	No restriction [91]	nr
miR-28-3p	nr	Inhibits viral transcription by targeting gag/pol mRNA [93]	nr
CIITA	Inhibits Viral trascription by targeting Tat and by recruiting RF in specific bodies [44, 101, 105]	Inhibits viral replication by targeting Tax-1 [106]	Inhibits viral replication by targeting Tax-2 [102, 103]

*nr* not reported

^a^References number

As A3G and other members of AG family, SAMHD1 acts at the early phase of HIV replication cycle prior to proviral integration, by diminishing the deoxynucleotide triphosphate pool, thus affecting viral reverse transcription [16, 28]. SAMHD1 has a triphosphohydrolase activity that is prevented in HIV-2 and related Simian immunodeficiency Viruses (SIV)s by the viral proteins Vpr and Vpx, respectively. SAMHD1 was originally described as a factor whose mutations are associated with an autoimmune conditions designated Aicardi–Goutières syndrome (AGS) with clinical manifestations resembling congenital viral infection and characterized by a high expression of type I interferon (IFN) and upregulation of IFN-stimulated genes [29]. Indeed, as the majority of RF, SAMHD1 is inducible by type I IFN in monocytes, and expressed at high levels in cells of myeloid origin and in resting CD4^+^ T cells that are refractory to HIV-1 infection [16, 28, 30].

Tetherin, also known as Bone Marrow Stromal Cell antigen 2 (BST-2) anchors budding viral particles at the late post-integration stages of replication on the surface of infected cells, preventing the release of HIV-1 and other enveloped viruses [14, 15]. HIV-1 Vpu, HIV-2 Env and SIV Nef antagonize Tetherin activity [31]. Unlike A3G and SAMHD1 proteins, functions other than RF activities have not been described for tetherin [32].

Tripartite motif proteins (TRIMs) are an E3 ligase family critical in many cellular functions, including the regulation and coordination of innate immunity and antiviral responses. They are characterized by a conserved tripartite motif, known as “RBCC”, comprising 3 functional domains: a RING, responsible for the E3 ubiquitine-ligase activity of the protein, one or two B-box(es) and a coiled-coil (CC) domain. The integrity of the TRIM motif is essential for their homo-multimerization and cellular localization [33, 34]. Several TRIM proteins target viral proteins directly to limit DNA and RNA virus infection. These TRIM proteins employ distinct mechanisms to inhibit viral entry, replication or dissemination [34, 35]. TRIM5α is the prototype of TRIM proteins in intrinsic immunity; it counteracts the cross-species transmission of retroviruses. TRIM5α was originally discovered as important determinant of the resistance of monkey cells to HIV-1 infection. Indeed, rhesus monkey TRIM5α (rhTRIM5α), but not human TRIM5α, potently limits HIV-1 infection in Old World monkeys by targeting the viral capsid, thus preventing the uncoating of the viral pre-integration complex [36, 37]. Unlike other RFs, the activity of TRIM5α is not antagonized by an accessory viral protein, since HIV-1 had evolved its capsid to avoid recognition by human TRIM5α, although it is still susceptible to the rhesus monkey version [38, 39]. Rhesus TRIM5α restricts a broad range of retroviruses including HIV-1, HIV-2, N-tropic murine leukemia virus (N-MLV), and equine infectious anemia virus (EIAV). Several other TRIM proteins also exhibit intrinsic antiretroviral activity, including TRIM11, TRIM28, TRIM19 and TRIM22 [34, 35]. Like TRIM5α, TRIM11 restricts HIV-1 reverse transcription by promoting premature viral uncoating. TRIM28 limits HIV-1 by binding the acetylated integrase, through the formation of a protein complex that includes the deacetylase HDAC1. TRIM19, also known as promyelocytic leukemia protein (PML), restricts the HIV-1 by inhibiting viral transcription [33]. Interestingy PML affects the stability of the HTLV-2 antisense APH-2 protein that is always expressed in HTLV-2 infected individuals and negatively regulates HTLV-2 transcription [40]. Thus PML may be also implicated in the control of HTLV-2 replication, although with a distinct effect. Of particular interest also TRIM22, similarly to TRIM5a, acts as RF against a broad spectrum of viruses. TRIM22 restricts HIV infection by two distinct mechanisms. First, it inhibits trafficking of gag protein to the plasma membrane, thus affecting the assembly of new viral particles [41]. Secondly, it acts as a transcriptional repressor of both basal and stimulated HIV-1 transcription induced by phorbol ester plus ionomycin, by preventing the binding of the cellular transcription factor Sp1 to HIV-1 promoter [42, 43]. Furthermore, we recently demonstrated that TRIM22 cooperates with the MHC Class II trasactivator (CIITA) to inhibit transcription initiation and elongation of the viral genome [44, 45] strongly suggesting that the combined action of the two factors may not only restrict viral replication but also potentially contribute to the establishment of viral latency [46, 47]. Besides inhibiting HIV-1 transcription, TRIM22 also inhibits Influenza A virus [48], Hepatitis B and C viruses [49, 50] and encephalomyocarditis virus [51], by using different mechanisms.

More recently, the family of potential RFs against HIV has substantially increased, indicating how much we still have to unveil about the complexity of molecules involved in intrinsic immunity [52].

## Intrinsic restriction targeting HTLV-1

As described above, the discovery of RFs and the description of their mechanism of action have been mostly derived by studies on HIV-1 retrovirus. The other extremely important member of human retroviruses affecting human health is HTLV-1, the first described human retrovirus, and the etiologic agent of a severe and still untreatable form of adult T cell leukemia/lymphoma (ATL) [53, 54]. HTLV-1 retrovirus is a member of an extended family of similar retroviruses, designated HTLV-2, HTLV-3 and HTLV-4 whose pathogenicity for humans is still unclear [55].

HTLV-1 infects approximately 10–20 million people world-wide, with high prevalence in the South of Japan, in sub-Saharan Africa, the Caribbean islands. Infection is also common in some regions of South America, the Middle East and Austro-Melanesia [56]. HTLV-1 induces clonal proliferation of infected cells to enhance its transmission, primarily by cell-to-cell contact [57]. Indeed cell-free HTLV-1 virus shows poor infectivity with the possible exception of dendritic cells (DCs) [58, 59] particularly if the virus is under the form of viral biofilms [60, 61]. Besides the aggressive malignancy of CD4+ T cells, HTLV-1 infection induces in 3–7% of subjects chronic inflammation processes including a serious and progressive neurological disease designated HTLV-1-associated myelopathy/tropic spastic paraparesis (HAM/TSP) [62, 63] as well as uveitis and dermatitis. Two viral regulatory proteins, Tax-1 and HTLV-1 basic zipper protein (HBZ), encoded by the sense and antisense viral transcripts, respectively, are thought to play key roles in HTLV-1 infection and disease progression [64, 65]. Tax-1 promotes viral transcription, and by deregulating several cellular pathways is considered responsible for the onset of neoplastic transformation [66]. Indeed, Tax-1 immortalize T cells and induces tumors in mice [67–69]. Interestingly, Tax-1 expression is frequently lost in ATL by either genetic or epigenetic modification of *tax* gene [70] and by the emerging immune response mediated by Tax-1-specific Cytotoxic T-Lymphocytes (CTL) [71]. Conversely, HBZ is ubiquitously expressed in ATL patients and HTLV-1 infected individuals, and HBZ mRNA abundance positively correlates with HTLV-1 proviral load (PVL) in asymptomatic carriers (AC), HAM/TSP and ATL patients [72]. Cumulatively, these observations suggest that Tax-1 exerts its oncogenic function early during ATL development, while HBZ may play a role in ATL maintenance and disease progression [65]. Recent studies on the comparative expression of endogenous Tax-1 and HBZ proteins in infected cells have highlighted specific distinctions that may bear additional importance on the role of these viral factors in HTLV-1-associated pathogenesis. While Tax-1 seems to be expressed both in the cytoplasm and in the nucleus in the early phases of infection and in HAM/TSP patients, HBZ expression clearly shows an exclusive cytoplasmic localization both in asymptomatic carriers and in HAM/TSP patients. Interestingly, in ATL, HBZ localization is predominantly seen in the nucleus, strongly suggesting that the pattern of expression and localization of this viral protein, more than Tax-1, could be used as markers of disease progression [73–75].

Cumulatively, these studied may in part explain the behavior of the classical adaptive immune response against HTLV-1, which is predominantly mediated by a strong CTL response against Tax-1 in the first phases of infection. Such response is believed to counteract virus spreading. Silent infection can proceed for decades. The subsequent adaptation and/or escape of the virus from host immunity is associated to down regulation of Tax expression and low response against HBZ [76, 77] as it is found during the progression of the infection toward neoplastic transformation. Within this complex picture of infection evolving versus an asymptomatic carrier state, a chronic neuroinflammatory process or neoplastic transformation, mechanisms of intrinsic immunity may certainly play a role. However, large studies of restriction factors involved in the control of HTLV-1 infection are still quite limited and in part controversial. Below we describe the studies that point to the involvement of the various families of RFs in the HTLV-1-host interaction.

## HTLV-1 and APOBEC3 family

As mentioned before, differently from HIV-1, HTLV-1 produces only very low levels of cell-free infectious virions, since it is transmitted by cell-to-cell contact. Interestingly, the genetic diversity of HTLV-1 is much lower than that of HIV-1 [78] even though both viruses target primarily A3G-expressing cells and despite the fact that HTLV-1 does not express an accessory protein analogous to HIV-1 Vif. Nevertheless, HTLV-1 seems to be relatively resistant to human APOBEC3 proteins since A3G-triggered G-to-A mutations were not detected in the proviruses from HTLV-1-infected patients, and only 0.1% of proviruses contained G-to-A hypermutations, suggesting that packaging of A3G into viral particles per se may not be sufficient to inhibit viral infectivity [24]. In line with these findings, another study showed that HTLV-1 was weakly susceptible to human A3G activity, despite A3G was efficiently encapsidated in HTLV-1 virions [25]. Conversely, another study showed that overexpressed as well as endogenous A3G incorporated into HTLV-1 virions inhibited the infection of HTLV-1 [79]. Derse et al. [26], explained this apparent A3G paradox, showing that the amount of A3G molecules packaged into HTLV-1 particles was less when compared to that incorporated in Vif-defective HIV-1 virus. This effect is caused by elements in the C-terminus of gag, which excluded human A3G from the HTLV-1 viral particles. When these elements were deleted or mutated, HTLV-1 was more susceptible to A3G inhibition and incorporated more A3G than wild-type virus into newly packaged virions. In ATL and asymptomatic carriers (AC) it has been hypothesized that non-sense mutations in viral genes induced by A3G might allow the virus to escape the host immune response. In addition, the fact that the target sequences of A3G were less frequent in *HBZ* coding region than in other genes, such as *tax*, may in part explain why HBZ is constantly expressed in ATL and during HTLV-1 infection [27]. The relative RF function of APOBEC3 may be influenced also from its level of expression. It was recently reported that the expression level of different APOBEC3 enzymes was similar in HAM/TSP patients and healthy donors, while there was an increase of A3B, but not A3G, in a model of HTLV-1 infected humanized mice [80]. Based on these data, the authors suggested possible implications of A3B upregulation in the susceptibility to HTLV-1 infection, although a direct involvement in HTLV-1-associated diseases could not be demonstrated. Interestingly, more recently A3B increased expression in both ATL and AC carriers has instead been reported by Kataoka et al. [81] in a very elegant study on integrated molecular analysis including whole-genome, exome and trascriptome sequencing of a large sample of ATL patients suggesting an implication also in HTLV-1 associated ATL. These findings are interesting because increased levels of A3B have been found in other tumor virus infections, such as those caused by HBV and HPV, suggesting a possible common mechanism of restriction for specific APOBEC3 RFs against oncogenic viruses [82, 83].

## HTLV-1 and TRIM family

Comparatively less information is available for other families of RFs other than APOBEC in HTLV-1 infection and associated diseases.

Recently, Leal et al. [84] by using a genome wide microarray analysis, compared levels of HTLV-1 PVL, the mRNA expression levels of Tax and HBZ with the mRNA expression of well-known anti-HIV-1 RFs. The comparison was done in healthy controls, HTLV-1 infected individuals and HAM/TSP patients. Of note, the authors identified a significant negative correlation of some host factors including TRIM5α, TRIM22 and tetherin/BST-2 with viral markers and clinical status. This negative correlation was found for example between certain polymorphisms of TRIM5α and HAM/TSP. Interestingly the very same polymorphisms were associated to high PVL, suggesting that variations in TRIM5α could be implicated in HTLV-1 replication [85]. It is of note that in the HIV-1 infection, allelic variants affecting coding sequences of another member of the TRIM family, TRIM22, have been linked to differential outcomes of HIV-1-associated pathology [86]. Among TRIM family members, it was demonstrated that also TRIM19/PML interferes with HTLV-1 replication by directing SUMOylated Tax-1 to PML nuclear bodies, thus causing its proteasomal degradation [87].

## HTLV-1 and SAMHD1

Although HTLV-1infects preferentially T cells, also cells of myeloid lineage, which play critical roles in the host innate response against viral infection, are targeted by HTLV-1 [58, 60, 88]. In the case of HIV-1, viral restriction in myeloid cells is in part mediated by SAMHD-1, which prevent productive DNA synthesis, thus limiting viral infection. On the other hand, SAMHD-1 antiviral function in HTLV-1 is controversial. Gramberg et al. [89], demonstrated that HTLV-1 is resistant to SAMHD-1 mediated-restriction. In contrast, other investigators have shown that HTLV-1 infection induces SAMHD-1-mediated apotosis in human primary monocytes through the recruitment of the cellular factor STING [90]. Thus, further studies are certainly required to finally assess whether SAMHD1 may or may not exert restriction function on HTLV-1.

## HTLV-1 and tetherin/BST-2

Studies related to a possible effect of tetherin, also called BST-2, on HTLV-1 infectivity have indicated that, unlike HIV-1, HTLV-1 does not express a protein to downregulate the expression levels of tetherin to overcome its restriction. Indeed, tetherin is highly expressed in chronically HTLV-1-infected cells and colocalizes with viral particles at the site of cell to cell contact. Nevertheless, silencing of tetherin impacts only minimally on infectivity of HTLV-1 although cell-to-cell transmission is certainly more relevant for HTLV-1 spreading as compared to HIV [91]. Based on this unique study it seems clear that tetherin does not affect the dissemination of the virus. Further studies are certainly needed to clarify the role of tetherin in HTLV-1 restriction.

## HTLV-1 and miR-28-3p

In recent years, several studies have shown the importance of micro RNAs (miRNA) in HTLV-1 infection and associated disease pathogenesis [92]. Interestingly one of this miRNAs, namely miR-28-3p, has been found to target a sequence localized within the viral gag/pol HTLV-1 mRNA. As a consequence, miR-28-3p reduced viral replication and gene expression. Indeed, cells expressing high level of miR-28-3p were found to be resistant to HTLV-1 infection [93]. These results are consistent with the observation that resting T cells, expressing high levels of miR-28-3p, are in fact relatively resistant to HTLV-1 infection as compared to activated T cells [93]. These obsevations justify the the designation of miR-28-3p as a new restriction factor for HTLV-1.

## HTLV-1 and CIITA

Another host factor endowed with anti-viral function for HTLV-1 is the MHC class II transcriptional activator, originally discovered in our laboratory as the major coordinator of expression of all MHC class II genes [94–96], By promoting the transcription of all MHC class II genes, the MHC class II transcriptional activator, also designated CIITA [97, 98], controls antigen presentation to CD4+ T helper (TH) cells, thus playing a critical role in the triggering of the adaptive immune response against a wide variety of antigens including pathogens [99]. CIITA is expressed constitutively in B cells and can be induced in human T cells upon activation with antigen or polyclonal stimuli, and in mielomonocytic cells under stimulation with interferon γ (IFNγ) [4]. The distinct mode of expression of CIITA is regulated by the activation of its different promoters. Promoter III is mostly responsible for the constitutive expression in B cells and for the expression in activated T cells; promoter I is mostly used for the expression in dendritic cells; and promoter IV is mostly responsible for the IFNγ-stimulated CIITA expression in myeloid and non-hematopoietic cells [100].

Besides its prominent role in the regulation of adaptive immune response, the first evidence that CIITA may act as an RF emerged in the context of HIV-1 infection, when we found it was acting as negative transcriptional regulator of HIV-1 expression in T cells. Here, CIITA inhibited virus replication by competing with the viral transactivator Tat for the binding to the Cyclin T1 subunit of the positive transcription elongation complex (P-TEFb) [101]. More recently we found that CIITA exerts its anti-viral function on HIV-1 by acting in concert with TRIM22, at least in myeloid cells [44, 45]. CIITA, like TRIM22, was expressed in HIV-1 poorly permissive U937 myeloid cell clones, and absent in the HIV-1-permissive U937 myeloid parental cells [44]. Importantly, as for TRIM22, the ectopic expression of CIITA in HIV-1-permissive U937 clones resulted in the inhibition of Tat-dependent HIV-1 replication, demonstrating the repression activity of CIITA also in myeloid cells. Of interest, the concomitant expression of CIITA and TRIM22 was required for the fully effective HIV-1 restriction observed in poorly permissive cells, suggesting that these two RF may cooperate to exert their antiviral function. In line with this hypothesis, we have recently reported that TRIM22 and CIITA are recruited in nuclear bodies also containing TRIM19/PML and Cyclin T1. These newly described nuclear bodies can be the first evidence of the existence of a concerted action of distinct restriction factors that, by convening in the same place, can synergistically counteract viral replication [44, 45].

**Fig. 1:**
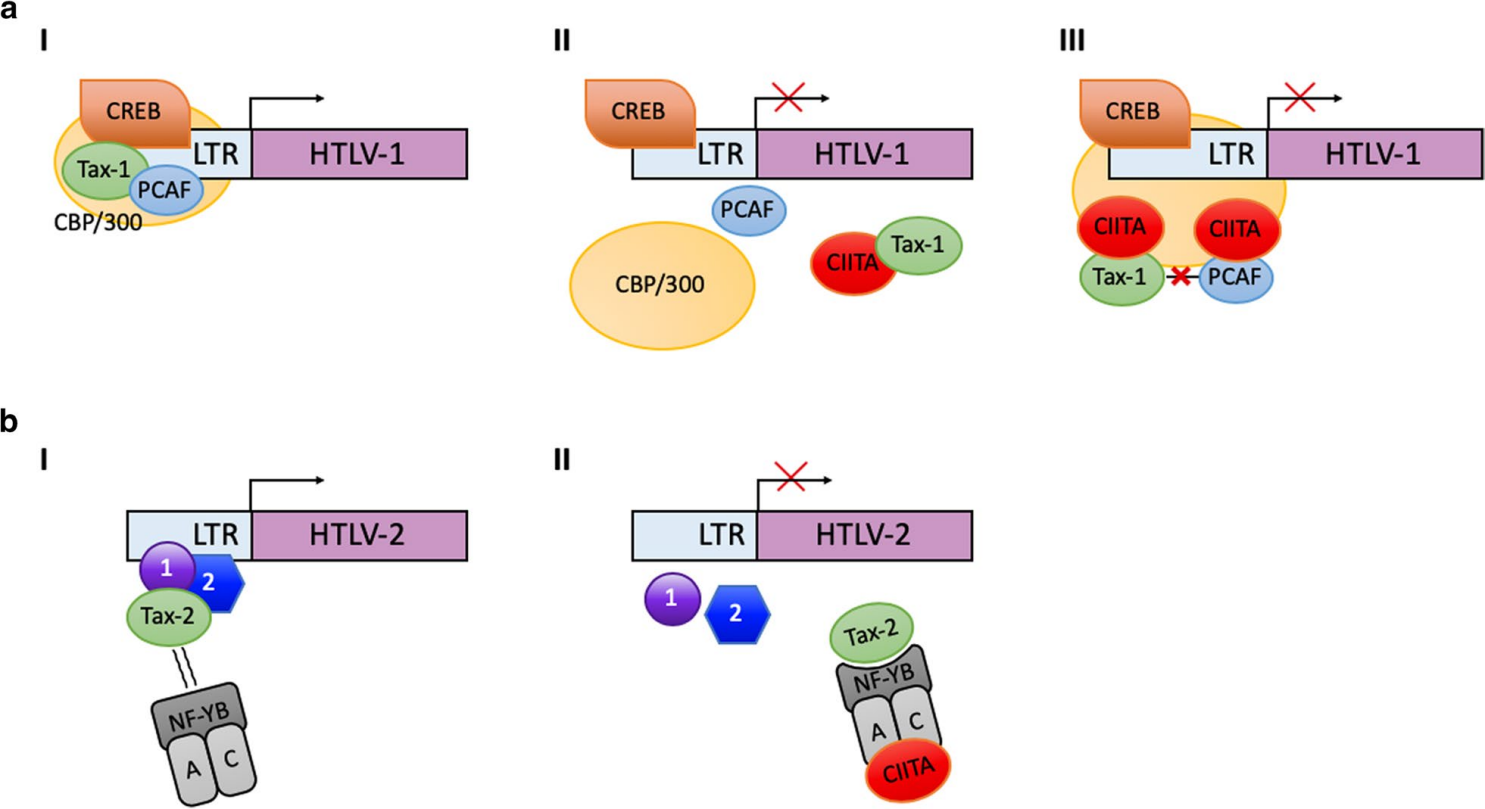
Possible mechanisms of CIITA-mediated inhibition of Tax-1-mediated and Tax-2-mediated LTR transactivation. **a** CIITA-Tax-1 association may impair in various ways Tax-1-mediated proviral transcription. aI In the absence of CIITA, Tax-1 promotes proviral genome transcription by inducing the formation of a multiprotein complex containing CREB, CBP and PCAF on the viral LTR promoter. aII In presence of CIITA, Tax-1 is bound by the MHC class II transactivator, preventing the physical formation and assembling of the multiprotein complex on the viral promoter, resulting in inhibition of LTR transcription. aIII Alternatively, Tax-1 in presence of CIITA can still be recruited on the viral LTR promoter with an assembled multiprotein complex which however is still not functional likely because the binding of Tax-1 to PCAF is inefficient due to steric hinderance generated by the Tax-1-CIITA interaction and/or PCAF-CIITA interaction. **b** In absence of CIITA, Tax-2 may bind endogenous NF-Y transcription factor but this binding is not sufficient to inhibit activation of HTLV-2 LTR and consequent proviral transcription (bI). In presence of CIITA, the NF-Y-CIITA complex strongly increases the affinity of NF-Y for Tax-2 thus recruiting Tax-2 and displacing it from the HTLV-2 LTR promoter. As a consequence, inhibition of HTLV-2 LTR transcription occurs (bII)

The first evidence that CIITA exerted an inhibitory function also on HTLV retroviruses dates back to 2004 when we demonstrated that CIITA blocks HTLV-2 virus replication both in T cells and B cells [102]. That CIITA was the unique responsible of the inhibitory effect was demonstrated by using two isogenic clones of B cells, consisting of CIITA-positive Raji cells and its CIITA-negative derivative RJ.2.2.5 [94]. After HTLV-2 infection, RJ.2.2.5 sustained very high levels of virus replication, whereas no relevant replication was observed in Raji parental cells. Consistent with this observation, the ectopic expression of CIITA in the permissive RJ2.2.5 cells resulted in a strong inhibition of HTLV-2 replication [102]. The molecular mechanism underlying this effect was rather complex as it involved the synergistic action of CIITA and NF-Y to displace Tax-2 from its interaction with cellular factors required to activate HTLV-2 promoter triggering [103] (Fig. 1b). These results prompted us to investigate whether the strongly pathogenic member of the HTLV family, HTLV-1, could also be affected by CIITA. Indeed, we could demonstrate that CIITA acts as a potent transcriptional repressor for HTLV-1. Again, by using both classical transfection models by which CIITA and HTLV-1 plasmid clones were transfected into 293T cells, and more importantly the isogenic promonocytic U937 cells, previously characterized for their efficient or inefficient capacity to support productive HIV-1 infection [104] and later shown not expressing or expressing endogenous CIITA, respectively, we observed that physiologic levels of CIITA efficiently inhibited HTLV-1 replication. In particular it was shown that, similarly to what observed with the HIV-1 infection [105], HTLV-1 replicated in the CIITA-negative HIV-1 permissive cells but not in the CIITA-positive poor permissive cells [106]. Importantly, the ectopic expression of CIITA in HIV-1 permissive U937 clones resulted in the inhibition of HTLV-1 replication [106]. As mentioned above, these clones express also TRIM22 and for HIV-1, we found that both CIITA and TRIM22 may contribute to the inhibition of HIV-1 replication [44, 45]. The potential interplay between CIITA and TRIM22 in this clonal model is presently under investigation also in the context of HTLV-1 infection. In searching for the molecular mechanism responsible for the CIITA-mediated HTLV restriction, we found that, as for HTLV-2 and for HIV-1, CIITA targets the major viral transactivator, in this case named Tax-1 [106]. Interestingly the molecular mechanism exerted by the CIITA to block the function of Tax-1 was different from the one invoved in Tax-2 blocking. Tax-1 and Tax-2 interact with several cellular factors, involved in many pathways of transcriptional activation and/or repression [107]. Remarkably, most of them, such as the above mentioned transcription factors NF-YB, the Histone Acetyl Transferases (HATs) p300, CBP, and PCAF, are also used by CIITA to promote MHC class II gene transcription [107]. HTLV-2 Tax-2 binds both endogenous and ectopically expressed NF-YB [103] however over-expression of NF-Y significantly inhibited Tax-2-driven HTLV-2 LTR transcription. Conversely neither endogenous nor over-expressed NF-Y could affect Tax-1-driven LTR transactivation [106]. Instead, we found that overexpression of PCAF, but not of p300, counteract the inhibitory action of CIITA on Tax-1, restoring transactivating function of the viral protein. Moreover, we demonstrated that CIITA, by binding to both PCAF and Tax-1, decreased the in vivo association of Tax-1 to PCAF [106]. Thus, CIITA might bind to and sequester PCAF from the transcriptional complex on the viral LTR promoter (Fig. 1aIII). Alternatively, CIITA by interacting with Tax-1 may simply prevent the association between PCAF and the viral transactivator (Fig. 1aII, III). In line with the hypothesis that CIITA could interfere with the recruitment of crucial host transcription factors on viral promoter, we also demonstrated that the overexpression of CREB and ATF1, both required for the assembly of the functional complex necessary for Tax-1 activation of HTLV-1 LTR promoter, counteracted the inhibitory action of CIITA on Tax-1 [106]. Thus, a general picture emerged suggesting that CIITA may exert its antiviral function against HTLV-1 by inhibiting the physical and functional interaction between the viral transactivator and crucial cellular factors needed to promote Tax-mediated HTLV-1 LTR transactivation.

## CIITA: a viral restriction factor inhibiting HTLV-1 oncogenicity?

**Fig. 2:**
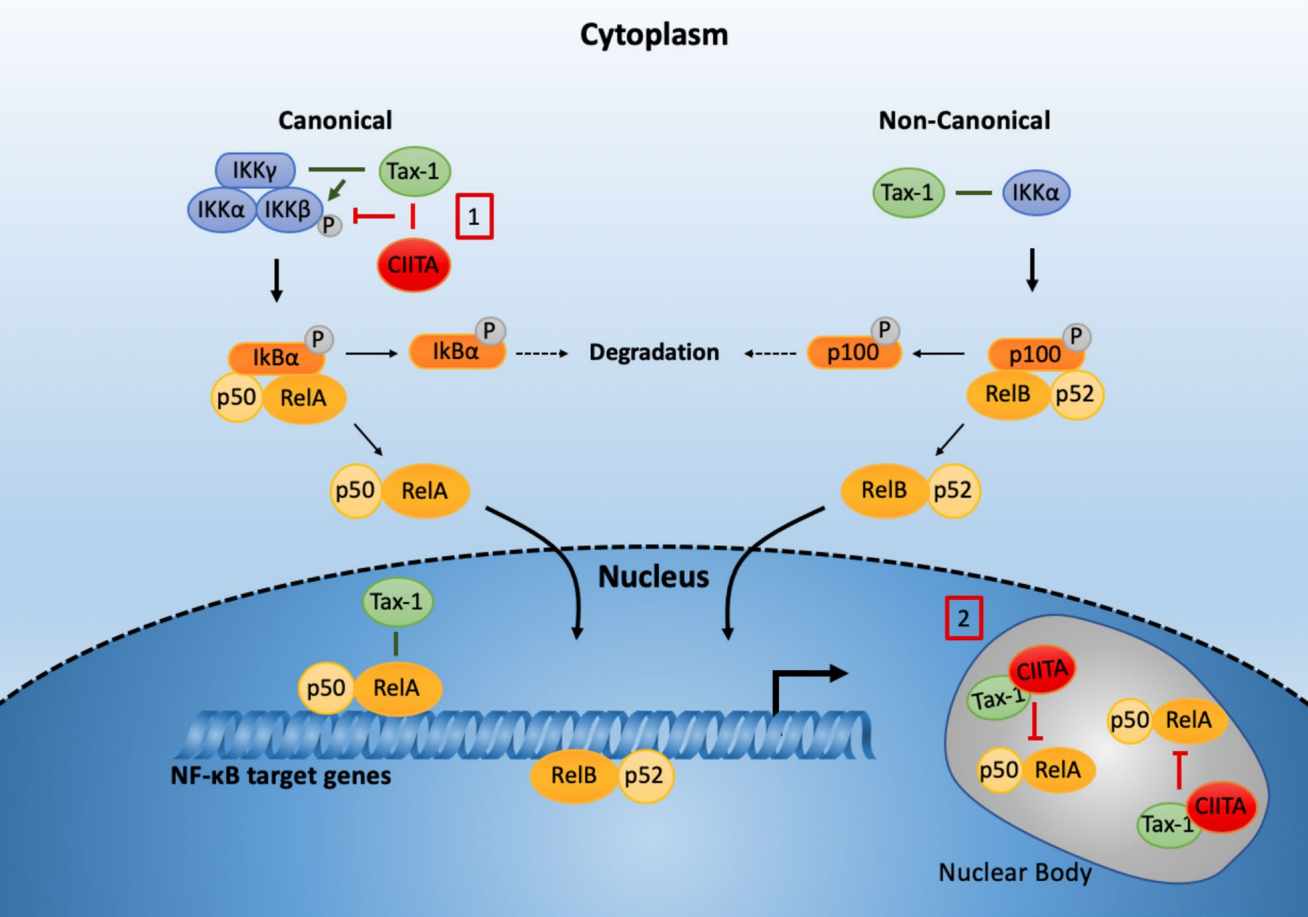
CIITA inhibits the Tax-1-mediated activation of the canonical NF-kB pathway. The oncogenic potential of Tax-1 is mostly due to its ability to constitutively activate NF-kB pathways. Tax-1 deregulates both the canonical and the noncanonical NF-kB pathway, by acting at different levels. In the canonical pathway, Tax-1 interacts with the gamma (γ) subunit of the trimeric IkB kinase (IKK), and activates IKK complex. The activated IKK phosphorylates IkB inhibitor bound to p50/RelA NFκB heterodimer. Following phosphorylation, IkB is degraded and the p50/RelA NF-kB complex migrates into the nucleus activating NF-kB target genes. In the non-canonical pathway, Tax-1 interacts and activates IKKα, which phosphorylates the inhibitory p100 subunit, thus inducing the activation and migration of the p52-/RelB NF-kB heterodimer into the nucleus. Moreover, Tax-1 promotes NF-kB activation in the nucleus by interacting with RelA and stabilizing the binding of p50/RelA to NF-kB-responsive promoters. CIITA exploits different strategies to suppress Tax-1-mediated NF-kB activation by acting in the nucleus and in the cytoplasm. In the cytoplasm [1], CIITA interacts with Tax-1 and this association does not prevent Tax-1 binding to IKKγ subunit of the IKK complex. Nevertheless, CIITA affects Tax-1-induced IKK activity, causing retention of the inactive p50/RelA/IkB complex in the cytoplasm. In the nucleus [2], nuclear CIITA associates with Tax-1/RelA in nuclear bodies, blocking Tax-1-dependent activation of NF-kB-responsive genes [2]

The great plasticity of CIITA molecule and the different mechanisms exerted by this host factor to counteract retroviral infections has been further confirmed and extended by our recent findings that CIITA binds directly to HTLV-1 Tax-1 [108]. Besides promoting proviral transcription, Tax-1 is a pivotal player in HTLV-1-induced T cell transformation [66]. Tax-1 exerts this function by modulating the expression of cellular genes and deregulating cell signaling pathways involved in cellular proliferation, such as the NF-kB pathway. We found that the persistent activation of the canonical NF-kB pathway by Tax-1 is strongly inhibited by CIITA not only in cells ectopically expressing CIITA, but more importantly in cells expressing endogenous CIITA [108]. Furthermore, mutant forms of CIITA constructed to be expressed in the nucleus or in the cytoplasm [106] have revealed that CIITA exploits different strategies to suppress Tax-1-mediated NF-kB activation both in the nucleus and in the cytoplasm (Fig. 2). Nuclear CIITA associates with Tax-1/p65-RelA and retains these factors in CIITA-containing nuclear bodies, thus blocking Tax-1-dependent activation of NF-kB-responsive genes [108]. Moreover, cytoplasmic CIITA traps Tax-1 in the cytoplasm, thus affecting Tax-1-mediated NF-kB p65-RelA heterodimer migration into the nucleus [108]. Part of this mechanism can be due to the fact that CIITA inhibits Tax-1-induced phosphorylation of IkB, suggesting a defective kinase activity of IKK complex. This result supports the idea that, in the presence of CIITA, IkB retains p65/RelA in the cytoplasm. Of note, the ability of CIITA to suppress IKK function did not correlate with an impaired association between Tax-1 and the IKKγ subunit of the IKK complex [108]. These findings are in line with the possibility of the formation of a trimolecular complex between CIITA, Tax-1 and IKKγ in which the latter is unable to activate the catalytic IKKα and IKKβ enzyme subunits of IKK complex, by steric hinderance or because it detaches from the complex. Whatever the mechanism, it is clear that these multiple inhibitory effects exerted by CIITA on the Tax-1-mediated activation of one of the crucial activation pathways involved in cell homeostasis may be of importance in counteracting the initial phases of oncogenic transformation that follow HTLV-1 infection.

## Conclusions

Although several studies have focused at identifying restriction factors and elucidate their antiviral mechanisms on HTLV-1 infection, much investigation is still required to delineate a structured framework similar to the one described for the RFs in HIV-1 infection. Controversial informations are part of this still reduced knowledge that results from an intrinsic limitation in studying HTLV-1-infected cells as compared to HIV-1-infected cells, due to the time frame through which HTLV-1 infection develops, the mode of viral transmission and the distinct pathological outcomes of infection. Nevertheless, some evidence of objective involvement of RFs in the control of HTLV retrovirus life cycle exists as well as preliminary important distinction on the putative mechanism of these RFs with respect to their mechanisms of action against HIV-1 (Table 1). This is the case for example of members of APOBEC family. HTLV-1, unlike HIV-1, does not have a Vif-like protein to counteract A3G enzymatic activity thus uses another escape mechanism to overcome the response of the host. HTLV-1 exploits A3G enzymatic activity to induce specific mutations in genes, such as Tax-1, against which the CTL response is very strong during the early phases of infection. This can limit the CTL recognition and thus the function of part of the adaptive immune stystem. On the same time A3G does not affect the HBZ gene, thus it is very likely that A3G is not involved in protection from disease progression and maintenance of neoplastic state. More obscure appears the role of A3B as HTLV-1 RF. Its increased expression in ATL and in AC as well as in other tumor virus infection may suggest a possible common role in infections of oncogenic viruses. Specific polymorphisms of the TRIM family of RFs, particularly TRIM5α and TRIM22, are associated to important variations in HTLV-1 proviral load, an event that has been correlated with the possible evolution of the infection toward the stronger susceptibility to HAM/TSP. Here certainly accurate studies are needed on the real mechanism of action of TRIM5α and TRIM22 in HTLV-1 infected cells to assess whether structural variation of these RFs is pathogenetically relevant or simply neutral associated marker of disease evolution.

As far as CIITA, its restricted tissue distribution to lymphoid and myelomonocytic cells, both susceptible targets of HTLV-1 infection, in conjunction with its inducible expression by IFNγ, similar to other RFs, and its strong inhibitory activity on HTLV-1 as well as HTLV-2 and HIV-1 (Table 1), makes it a peculiar RF whose potential use to counteract viral replication and spreading against retroviruses must be still fully appreciated. It should not be underestimated that in chronic infections as HTLV-1 infection, by keeping down the replication of the virus CIITA may also contribute to establishing a state of proviral latency. Finally, the CIITA peculiar action on HTLV-1 Tax-1 as inhibitor of the viral transactivator constitutive activation of the NF-kB pathway involved in the onset of oncogenic process, makes it a potential biological weapon to counteract oncogenic transformation in HTLV-1 infected cells. At present CIITA is the only factor that combines two crucial function of immunity: the control of adaptive immunity via its role on the expression of MHC class II genes and thus on antigen presentation, and its function as restriction factor against retroviruses. This dual role against pathogens during evolution is exceptionally unique and certainly warrant future fascinating studies.

## Data Availability

Not applicable.

## Abbreviations

RF: restriction factor
IFN: interferons
HTLV-1: Human Leukemia/Lymphoma Virus type I
HIV-1: human immunodeficiency virus I
Vif: viral infectivity factor
Vpu: viral protein U
Nef: negative regulatory factor
TRIM: tripartite Motif
APOBEC3: apolipoprotein B mRNA editing enzyme-catalytic polypeptide-like 3
SAMHD-1: Sterile Alpha Motif and HD containing protein 1
HBV: hepatitis B virus
BST-2: bone marrow stromal cell antigen 2
N-MLV: N-tropic murine leukemia virus
EIAV: equine infectious anemia virus
CIITA: class II transactivator
PML: promyelocytic leukemia protein
DC: dentritic cells
AC: asymptomatic carriers
PLV: proviral load
ATL: adult T cell leukemia
HAM/TSP: HTLV-1-associated myelopathy/tropic spastic paraparesis
HBZ: HTLV-1 basic zipper protein
TH: T helper
